# “Unspecified organic personality and behavioral disorder due to brain damage from HHV-6 encephalitis in child. case report and literature review”

**DOI:** 10.1192/j.eurpsy.2023.357

**Published:** 2023-07-19

**Authors:** A. Oliva Lozano, M. A. Morillas Romerosa, P. Herrero Ortega, J. Garde Gonzalez, B. Orgaz Álvarez, J. Curto Ramos, M. Alcamí Pertejo

**Affiliations:** ^1^Psychiatry, Clinical Psychology and Mental Health, La Paz University Hospital; ^2^Psychiatry, Clinical Psychology and Mental Health, Hospital Universitario “La Paz”, Madrid, Spain

## Abstract

**Introduction:**

We present a case of a 15 year-old boy diagnosed with Unspecified Personality and Beheavioral Disorder Due to Brain Damage from a Human Herpes Virus-6 Encephalitis.

**Objectives:**

To describe a case of an Unspecified Organic Personality and Behavioral Disorder secondary to brain damage from Human Herpes Virus-6 (HHV-6) Encephalitis in an 11 year-old childand to review recent literature, in order to improve clinical practice.

**Methods:**

Clinical case report and brief review of literature. A bibliographic research was made in the database PubMed, using the terms “Viral Encephalitis” AND “Neuropsychiatric symptoms”; “Viral Encephalitis” AND “Behavioral Disorder”; “Long-Term Neurological Morbidity” AND “Viral Encephalitis”.

**Results:**

15 year-old boy diagnosed with Unspecified Personality and Beheavioral Disorder Due to Brain Damage from a Human Herpes Virus-6 Encephalitis, secondary to immunosupression in the context of haematopoietic progenitor transplantation (HPT) at 11 years old. MRI showed supratentorial ventriculomegaly, atrophic changes in encephalon and right hippocampus with subcortical retraction secondary to previous encephalitis. Clinically, main changes appeared in behavior, presenting a serious frontal syndrome with high disinhibition, what implied severe social and academic difficulties. During the outpatient follow-up, the behavioural disorder is being pharmacologically treated with Risperidone 1,5mg per day with a partially favorable evolution. The patient presented intolerance to olanzapine, with an episode of low level of conciuosness after taking it.

Bibliographic research results indicate that the gold standard treatment for behavioral disturbances are antipsychotics. Risperdidone is proven save for treatment in children. Results point out also the importance of an early multidisciplinar intervention, involving family training, rehabilitation resources and curricular adaptations.

**Image:**

**Figure:**
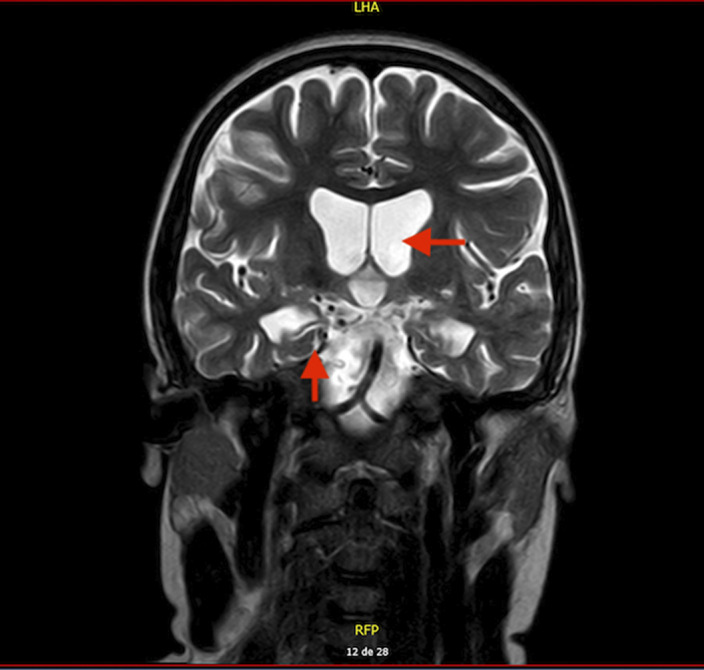

**Image 2:**

**Figure:**
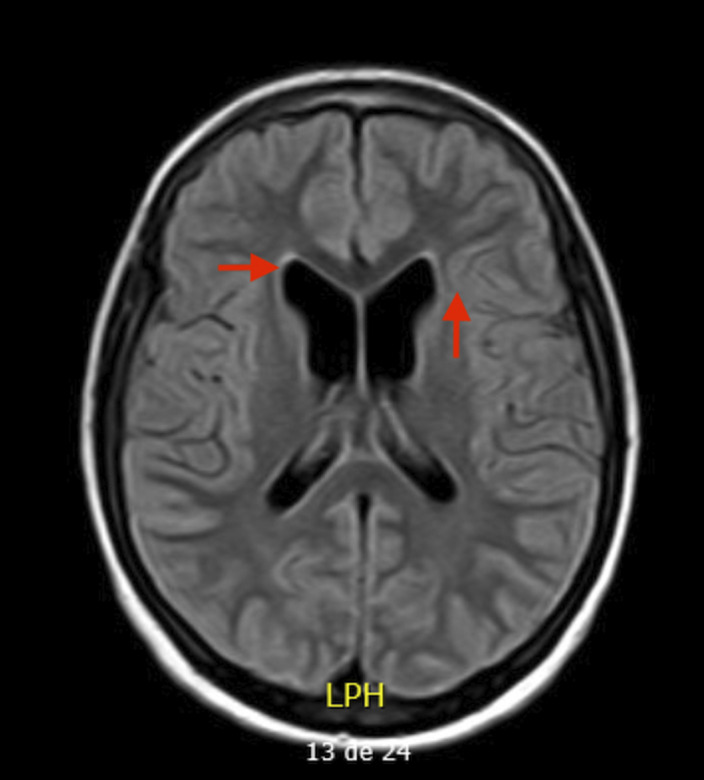

**Conclusions:**

Viral encephalitis may have serious neuropsychiatric consequences, especially during childhood while the brain development is not finished. When the neurological damage affects the frontal lobes of the brain, behavioural and personality disturbances are expected and an early multidisciplinar intervention should be considered. Antypsichotics are the gold standard pharmacological treatment for behavioural disturbances. During the scholar period, special curricular adaptations should be done in order to reduce study-related stress.

**Disclosure of Interest:**

None Declared

